# European wildcat populations are subdivided into five main biogeographic groups: consequences of Pleistocene climate changes or recent anthropogenic fragmentation?

**DOI:** 10.1002/ece3.1815

**Published:** 2015-12-07

**Authors:** Federica Mattucci, Rita Oliveira, Leslie A. Lyons, Paulo C. Alves, Ettore Randi

**Affiliations:** ^1^ Laboratorio di GeneticaIstituto Superiore per la Protezione e la Ricerca Ambientale (ISPRA)40064Ozzano dell'EmiliaBolognaItaly; ^2^ InBio ‐ Laboratório AssociadoCentro de Investigação em Biodiversidade e Recursos Genéticos (CIBIO)Universidade do PortoCampus de Vairão4485‐661VairãoPortugal; ^3^ Departamento de BiologiaFaculdade de Ciências da Universidade do Porto4099‐002PortoPortugal; ^4^ Department of Veterinary Medicine & SurgeryCollege of Veterinary MedicineUniversity of Missouri–ColumbiaColumbia65211MissouriUSA; ^5^ Wildlife Biology ProgramDepartment of Ecosystem and Conservation SciencesUniversity of MontanaMissoula59812MontanaUSA; ^6^ Department 18/Section of Environmental EngineeringAalborg University9000AalborgDenmark

**Keywords:** ABC simulations, Bayesian clustering, conservation genetics, *Felis silvestris*, microsatellites, phylogeography, population structure, wild and domestic cat hybridization

## Abstract

Extant populations of the European wildcat are fragmented across the continent, the likely consequence of recent extirpations due to habitat loss and over‐hunting. However, their underlying phylogeographic history has never been reconstructed. For testing the hypothesis that the European wildcat survived the Ice Age fragmented in Mediterranean refuges, we assayed the genetic variation at 31 microsatellites in 668 presumptive European wildcats sampled in 15 European countries. Moreover, to evaluate the extent of subspecies/population divergence and identify eventual wild × domestic cat hybrids, we genotyped 26 African wildcats from Sardinia and North Africa and 294 random‐bred domestic cats. Results of multivariate analyses and Bayesian clustering confirmed that the European wild and the domestic cats (plus the African wildcats) belong to two well‐differentiated clusters (average *Ф*_ST_ = 0.159, *r*_st_ = 0.392, *P* > 0.001; Analysis of molecular variance [AMOVA]). We identified from *c*. 5% to 10% cryptic hybrids in southern and central European populations. In contrast, wild‐living cats in Hungary and Scotland showed deep signatures of genetic admixture and introgression with domestic cats. The European wildcats are subdivided into five main genetic clusters (average *Ф*_ST_ = 0.103, *r*_st_ = 0.143, *P* > 0.001; AMOVA) corresponding to five biogeographic groups, respectively, distributed in the Iberian Peninsula, central Europe, central Germany, Italian Peninsula and the island of Sicily, and in north‐eastern Italy and northern Balkan regions (Dinaric Alps). Approximate Bayesian Computation simulations supported late Pleistocene–early Holocene population splittings (from *c*. 60 k to 10 k years ago), contemporary to the last Ice Age climatic changes. These results provide evidences for wildcat Mediterranean refuges in southwestern Europe, but the evolution history of eastern wildcat populations remains to be clarified. Historical genetic subdivisions suggest conservation strategies aimed at enhancing gene flow through the restoration of ecological corridors within each biogeographic units. Concomitantly, the risk of hybridization with free‐ranging domestic cats along corridor edges should be carefully monitored.

## Introduction

Past climate changes, historical evolutionary events and, eventually, more recent anthropogenic pressures shaped the partition of genetic diversity within and among populations (Hewitt 2000; Banks et al. 2013). Mammalian species adapted to temperate climates survived the Pleistocene glaciations into three main Mediterranean refuges in the southern Iberian, Italian, and Balkan peninsulas, from where they moved to recolonize central and northern Europe during the interglacials (Zachos and Hackländer 2011). This phylogeographic framework includes the postulated existence of cryptic northern refuges (Stewart and Lister 2001), complex patterns of refuges‐within‐refuge (Gómez and Lunt 2007), and the genetic consequences of secondary contacts and hybridization (Hewitt 2001). Recent anthropogenic actions (deforestation, over‐hunting, and the spread of domesticated and alien taxa) have deeply affected the underlying natural phylogeographic subdivisions. Conservation strategies to preserve and restore the historical biogeographic patterns should unravel natural and anthropogenic causes of genetic subdivisions. The use of molecular markers and powerful computational tools has provided unique ways for assessing species’ phylogeographic structure and promoting conservation strategies based on sound scientific knowledge (Hickerson et al. 2010). Phylogeographic frameworks help to delimit appropriate evolutionary and management units (ESU and MU; Funk et al. 2012) and identify genes causing local adaptations (Allendorf et al. 2010). In this study, we used the European wildcat, a mammalian mesocarnivore widely distributed across Europe, as a model to investigate the value of species’ phylogeographic structure for conservation planning.

The wildcat (*Felis silvestris*) comprises a number of poorly described subspecies that inhabit the entire Old World (Nowell and Jackson 1996). In Europe, three subspecies coexist: the European wildcat (*F. s. silvestris*, Schreber 1777), distributed from Portugal to Romania; the African wildcat (*F. s. libyca*, Forster 1780), in the Mediterranean islands of Sardinia, Corsica and Crete; and the domestic cat (*F. s. catus*). According to archeological remains, the European wildcat appeared in the continent around 450,000–200,000 years ago, but its fossil record was limited to the three southern Mediterranean peninsulas during the last glaciations (Sommer and Benecke 2006). The presence of African wildcats in Mediterranean islands is a much more recent consequence of human translocations at very early stages of domestication, less than 11,000 years ago, by Neolithic navigators. The earliest evidences of close cat–human relationships were found in Cyprus deposits from 10,600 years ago (Vigne et al. 2012), but real domestication processes likely began when humans started to build the first civilizations over the Fertile Crescent (Driscoll et al. 2007; Lipinski et al. 2008). Evidences for cat domestication are known from China (c. 5500 years ago) and Egypt (c. 4000 years ago; Hu et al. 2014). Domesticated cats promptly colonized the entire world and became very common in Europe, spreading via the major land and sea trade routes of Romans, Etruscans, and Greeks (Clutton‐Brock 1999; Lipinski et al. 2008). The sudden diffusion of free‐ranging domestic cats created the conditions for crossbreeding and introgression of domestic alleles into wildcats’ genomes, perhaps compromising the evolutionary trajectories of the European wildcat (Beaumont et al. 2001; Pierpaoli et al. 2003; Lecis et al. 2006; Oliveira et al. 2008a,b, 2015).

**Figure 1:**
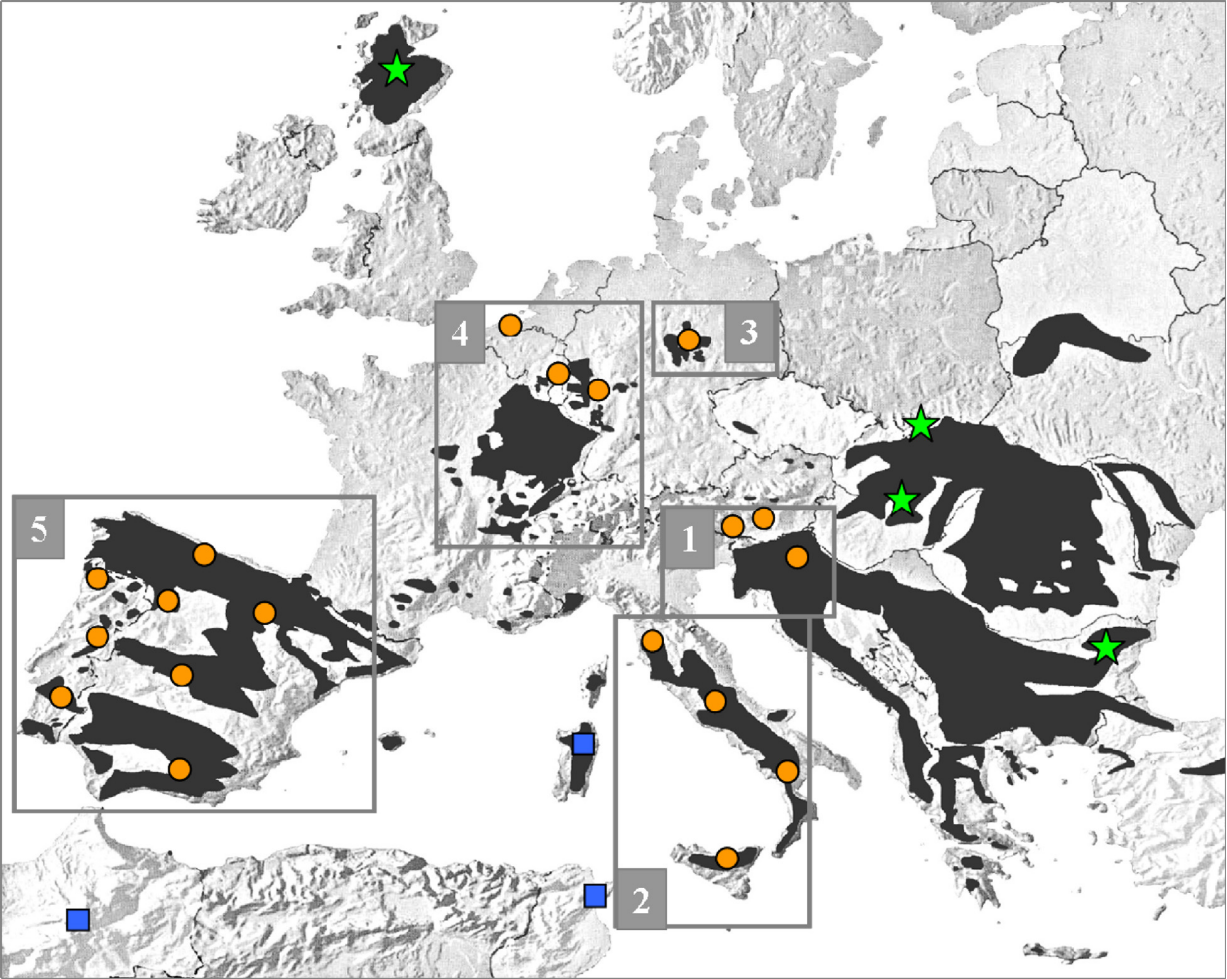
Approximate distributions and sampling locations of wildcats (*Felis silvestris*) collected across Europe and North Africa. Distributions are represented by dark areas (adapted from Grabe and Worel 2001). The five European wildcat (*F. s. silvestris*) biogeographic groups identified through multivariate and Bayesian cluster analyses are indicated by numbered squares. Star symbols indicate the approximate location of the admixed European wildcat populations in eastern Europe (Poland and Bulgaria), and the introgressed domestic (*F. s. catus*) × European wildcat population in Hungary and Scotland. Sampling regions of African wildcats (*F. s. libyca*) are indicated by square symbols (Morocco and Libya in north Africa; the Island of Sardinia in Italy).

European wildcat populations are fragmented throughout most of the central and western European countries (Fig. 1; Mitchell‐Jones et al. 1999), the likely consequence of recent anthropogenic events (deforestation, direct persecution, and local decline of major prey). However, with a few local exceptions in Italy (Mattucci et al. 2013), France (Say et al. 2012), Germany (Eckert et al. 2009; Hertwig et al. 2009), and Iberia (Oliveira et al. 2008a,b), the underlying patterns of genetic variability are unknown. European wildcats are associated mainly with broadleaved forests and their micromammal prey communities (Mattucci et al. 2013; and references therein), but viable populations also exist in Mediterranean ecosystems (Lozano 2010). In a previous study, we hypothesized that European wildcats survived the glacial periods from mid‐Pleistocene to the Holocene in a number of fragmented refuges (Mattucci et al. 2013). Pleistocene climatic changes could have shaped wildcat's continent‐wide partition of genetic diversity (Kitchener and Rees 2009). However, a comprehensive phylogeography of wildcats in Europe is still missing.

Here, we report the most comprehensive range‐wide study of European wildcat population structure that was designed aiming at reconstructing their main underlying phylogeographic patterns. We predicted that European wildcat refugial populations have survived the last glaciation in fragmented areas of broadleaved forest scattered around the Mediterranean and located mainly in the Iberian, Italian, and Balkan peninsulas. Consequently, the observed patterns of population structuring should have been generated during the last few thousand years, and not as recently as a few centuries, as predictable in case of recent anthropogenic fragmentation events. Thus, we aimed to (1) estimate the extent of genetic diversity within and between wild and domestic cat populations; (2) evaluate the patterns of population structuring and fragmentation in European wildcats; (3) identify genetic signatures of demographic fluctuations; and (4) obtain estimates of population divergence times. The evaluation of the genetic consequences of historical and recent fragmentations is helpful to define European wildcat conservation units and forecast their conservation perspectives.

## Material and Methods

### Sampling and laboratory procedures

A total of 1124 tissues, blood, saliva, hair, or skin samples from European wildcats (*Fsi*), domestic cats (*Fca*), and African wildcats (*Fli*) were opportunistically collected over a 12 year period (1998–2010; Fig. 1, Table S1). European wildcats, covering most of the species range in 15 European countries, were morphologically identified by collectors according to coat color patterns, cranial, and intestinal indexes (Schauenberg 1969, 1977; French et al. 1988; Ragni and Possenti 1996). Almost all the European wildcat samples were collected from found‐dead or trapped animals, likely very close to their individual home ranges. The domestic cat sample included free‐ranging or owned cats. The African wildcats were sampled in Sardinia (Italy) and North Africa (Morocco and Libya). Aiming to help the identification of hybrid cats, we added 17 previously described European wild × domestic cat hybrids from Italy. Seven hybrids were obtained in captivity by controlled crossings (Ragni 1993). The other ten wild‐living hybrids were genetically identified in other studies (Pierpaoli et al. 2003; Lecis et al. 2006; Mattucci et al. 2013) and reanalyzed here. Samples were always collected respecting rules on animal welfare, and no cat was killed to obtain samples.

Samples were stored at −20°C in 5 volumes of 95% ethanol (tissues, skins and hairs) or in Longmire et al. (1997) Tris/SDS buffer (blood, buccal swabs). Genomic DNA was extracted using the QIAGEN DNeasy tissue and blood kits (Qiagen Inc, Hilden, Germany). Thirty autosomal dinucleotide and one tetranucleotide (Fca 441) microsatellites (STR; Table S2), originally identified in domestic cats (Menotti‐Raymond et al. 2003) and screened in other domestic and wildcat studies (Lipinski et al. 2008), were amplified in eight PCR multiplex reactions using the Qiagen Multiplex PCR Kit (primer labeling, PCR recipes, and thermocycling protocol are reported in Table S2). Hair and skin samples were amplified in four replicates in dedicated UV‐hoods, following a multitube approach designed for low‐quality DNA samples. The amplicons were analyzed in an ABI 3130 XL DNA Analyzer (Applied Biosystems Inc., Foster City, CA), and allele sizes were calibrated with GeneScan‐500 LIZ and determined using GeneMapper 4.1 (Applied Biosystems Inc.). All extraction and PCR steps included negative controls (no DNA). A reference positive control (known genotypes) was always included to assess PCR success and calibrate independent sequencing runs.

The power of the chosen STR's panel to identify individual genotype profiles was evaluated by calculating the probability‐of‐identity values (PID and PIDsibs; Waits et al. 2001) in GenAlEx 6.41 (Peakall and Smouse 2006). About 10% of randomly selected samples were independently replicated twice to assess rates of allelic dropout and false alleles. The presence of null alleles was assessed with Microchecker (Van Oosterhout et al. 2004) with an adjusted *P*‐value corresponding to *Δ *= 0.05 after Bonferroni correction (Rice 1989). Individual profiles were matched to exclude replicates.

### Analyses of genetic diversity and differentiation

Genetic diversity was estimated separately for the domestic, African, and European cat subspecies, after excluding all cats from Scotland and Hungary and all the hybrids identified in preliminary admixture analyses (see below). Genetic diversity within each of the five European wildcats clusters identified by Bayesian structure analyses (see below) and also evaluated. We used Arlequin 3.5.1.2 (Excoffier and Lischer 2010) to: (1) estimate allele frequencies, mean number of alleles per locus (*N*_A_), observed (*H*_O_), and expected heterozygosity (*H*_E_); (2) test for deviations from Hardy–Weinberg equilibrium (HWE), with a Markov Chain length of 10^5^ and 3000 dememorization steps; (3) test for pairwise linkage disequilibrium (LD), with 100 initial conditions followed by 16,000 permutations, for all locus–population combinations, based on Guo and Thompson's (1992) exact test. The *P*‐values were adjusted for multiple tests using a sequential Bonferroni correction. Allelic richness for each population (*N*_AR_) was estimated following a rarefaction method that compensates for uneven sample sizes (Hp‐Rare; Kalinowski 2005). Genetic differentiation among subspecies and European wildcat clusters was estimated using with pairwise *f*_st_ (Weir and Cockerham's 1984) and *r*_st_ (Slatkin 1995) in Genepop 4.1 (Rousset 2008) and Fstat 2.9.3.2 (Goudet et al. 2002), respectively. Analysis of molecular variance (AMOVA) on Euclidean pairwise genetic distances was estimated using analogues of Wright's *F*‐statistics.

We tested for very recent bottlenecks (up to the first 10th generations ago) using the “heterozygote excess” and the “mode‐shift” procedure (Luikart et al. 1998) in Bottleneck 1.2.02 (Cornuet and Luikart 1997), assuming a microsatellite “two‐phase mutational model” with 95% one‐step mutations. Two‐tailed Wilcoxon signed rank test was used for determining the significance of the observed deviations. Less recent bottlenecks (up to a few hundred generations ago) were tested with Garza and Williamson's (2001) “*m‐*ratio test” in software M_P_Val. The values of *m* was computed as the ratio of the number of alleles (*k*) over their range in fragment sizes (*r*), which is predicted to decline in a bottleneck because the number of alleles should decrease faster than the range in fragment sizes. The significance of *m* was determined by comparison with critical values (*M*c), calculated from hypothetical populations in mutation‐drift equilibrium using the program Critical_M with 10,000 simulation replicates. We used a microsatellite “two‐phase mutation model” with an average size of multistep mutations Δ*g* = 3.5, assuming 90% stepwise mutations (*P*_s_), as recommended by Garza and Williamson (2001). We set *θ* = 5 or 10 (being *θ* = 4 Ne*μ*, where *Ne* is the effective population size and *μ* is the mutation rate) to evaluate the sensitivity of the method to this parameter.

### Population structure, assignment, and admixture analyses

Population genetic clusters were estimated using Structure 2.3.4 (Pritchard et al. 2000; Falush et al. 2007; Hubisz et al. 2009) with the “admixture,” “*F*,” and “*I*” models, both with or without prior nongenetic information (subspecies or geographic population of origin). We aimed to: (1) infer the number *K* of a‐priori unknown genetic clusters in the sample; (2) estimate the average proportion of membership (*Q*i) of the sampled populations to each cluster; and (3) assign each multilocus genotype to one or more cluster, according to their posterior individual probability of membership (*q*_i_). Based on previously published admixture analyses of observed and simulated cat datasets (Oliveira et al. 2008a; Randi 2008), we used a threshold *q*_i_ = 0.80 to assign the genotypes to the clusters. Each run was replicated five times, with 10^4^ burn‐in followed by 10^5^ MCMC iterations. The optimal number of clusters was identified using the Δ*K* statistics in CorrSieve 1.6.1 (Evanno et al. 2005; Campana et al. 2011). Results of the five replicates were averaged using Clump and Distruct procedures in Clumpak (http://clumpak.tau.ac.il).

All genotypes with possible hybrid ancestry were preliminary analyzed, using Structure with two different datasets to assign individuals to two populations (*K *= 2): European wildcats versus domestic cats, and African wildcats versus domestic cats. The analyses were replicated within each of the five European wildcat biogeographic clusters to overcome a possible bias due to within‐subspecies population structuring. Cats’ ancestry was computed using *K* = 2 with prior population information (option *usepopinfo* activated) for the domestic and wildcats that were genetically preidentified in the first runs of Structure. We subsequently excluded all the hybrids and the admixed cats from Scotland and Hungary. Then, we used a hierarchical approach to determine the divergence among the three cat subspecies and the five European wildcat clusters, assuming *K* from 1 to 15. We also explored the patterns of differentiation among cat subspecies and European wildcat clusters (excluding all hybrids) by Discriminant Analysis of Principal Components (DAPC) in the Adegenet package (Jombart 2008).

### Estimation of demographic changes and divergence times among European wildcat populations

Approximate Bayesian Computation simulations (ABC; Beaumont et al. 2002) implemented in the software popABC (Lopes et al. 2009) was used to model plausible evolutionary scenarios and estimate divergence times (in generations) among the European wildcat clusters identified by Structure. In order to compare alternative scenarios and estimate divergence times assuming that those groups diverged before the Last Glacial Maximum (i.e., before *c*. 20,000 years ago) or during the Holocene (i.e., less the last *c*. 12,000 years), we used popABC (REF). Three alternative population histories (Fig. S1) were modeled in each of the following datasets: (I) three population groups that could have originated during colonization–fragmentation events in central Europe, that is wildcats sampled from central European regions (Belgium, Luxembourg, western Germany), central Germany, and north‐eastern Alpine–Dinaric regions; (II) three population groups that could have originated in Pleistocene Mediterranean refuges: wildcats from the Iberian peninsula (Portugal and Spain), peninsular Italy (excluding Sicily), and north‐eastern Alpine–Dinaric regions (eastern Italian Alps, Austria, Slovenia, Croatia); (III) isolation in Sicily, comparing samples from peninsular Italy and Sicily. All simulations were modeled using the STR generalized stepwise mutation model (Goldstein and Pollock 1997), assuming an isolation with no migration model in which populations have diverged from a single ancestral population (Nielsen and Wakeley 2001). Three summary statistics (heterozygosity, variance in allele length and number of alleles) were simulated 500,000 times. The mutation rates for each of the 31 loci were drawn from a normal distribution with mean = 0.0001, standard deviation = 0, and mean of the standard deviation = 0.0005. We used prior population parameters with uniform distributions bound between minimum and maximum values. The parameters were estimated using 10,000 simulations (tolerance index = 0.02). Rejection steps were performed in *R* using scripts developed by M. Beaumont (http://code.google.com/p/popabc/model_choice.r) and modified to fit our analyses. We also used the (*δμ*)^2^ genetic distance (Goldstein et al. 1995b) and the equation (*δμ*)^2^ = 2*μT* (*μ* = mutation rate; *T* = generations; Goldstein and Pollock 1997) to infer divergence times among the European wildcat populations. We assumed that populations were at mutation‐drift equilibrium and had historically stable effective population size and that STR evolved at mutation rates *μ* = 5.6 × 10^−4^ (estimated by popabc) and *μ* = 2.05 × 10^−4^ (used in felid species by Driscoll et al. 2002).

## Results

### Genetic diversity

All the 31 microsatellites were polymorphic in the genotyped 668 presumptive European wildcats (*Fsi*), 26 African wildcats (*Fli*), 294 domestic cats (*Fca*), and 136 admixed cats from Hungary (*n* = 98), Scotland (*n* = 21), and Italy (*n* = 17; Fig. 1, Table S1). We did not detect any allelic drop‐out or false allele in 100 replicated genotypes nor find any identical genotypes. Genotype pairs mismatched at a minimum of two loci. The values of probability‐of‐identity were very low: PID = 2.7 × 10^−34^, PIDsibs = 4.8 × 10^−13^ in *Fsi*; PID = 1.6 × 10^−40^, PIDsibs = 1.2 × 10^−14^ in *Fli*; and PID = 2.0 × 10^−38^, PIDsibs = 4.9 × 10^−14^ in *Fca*, ensuring that distinct individuals should not have the same genotype by chance. Excluding the admixed cats, the allele numbers ranged across loci from *N*_A_ = 6 to 32, the observed and expected heterozygosities varied from *H*_O_ = 0.04 to 0.87 and from *H*_E_ = 0.06 to 0.91 (Table S2). The mean values of heterozygosity were not significantly different among the three cat subspecies (Table 1), which had lower than expected *H*_O_ values and significantly positive *F*_IS_ = 0.14 (*Fca*), 0.19 (*Fsi*), and 0.13 (*Fli*; all values with *P *< 0.001), suggesting the pooling of samples from genetically distinct populations within the same subspecies. The number of significant pairwise correlations between loci (testing for departure from LE) was zero in *Fli*, three in *Fca*, and 81 in the total *Fsi* sample, but much smaller in the five genetic clusters (Tables 1 and S2), a likely consequence of pooling samples from distinct genetic subpopulations.

**Table 1:** Variability at 31 autosomal microsatellites in three cat subspecies (domestic cat *F. s. catus*; African wildcat *F. s. libyca*; and European wildcat *F. s. silvestris*) and in five European wildcat biogeographic groups identified by Bayesian clustering analyses

Subspecies	Populations	Acronym	*N*	*N*_A_	*n*_ar_	*H*_O_	*H*_E_	*F*_IS_	HWE	LE
Domestic cats	All	*Fca*	293	15.3 (4.9)	9.6	0.68 (0.09)	0.79 (0.09)	0.14*	22	3
African wildcats	All	*Fli*	26	10.3 (2.6)	10.3	0.72 (0.10)	0.83 (0.05)	0.13*	2	0
European wildcats	All	*Fsi*	609	14.2 (3.1)	8.0	0.59 (0.17)	0.73 (0.19)	0.19*	30	81
	*Group 1*	*Fsi*‐1	141	9.8 (2.3)	7.7	0.63 (0.18)	0.69 (0.18)	0.09*	6	4
	*Group 2*	*Fsi*‐2	132	9.8 (2.2)	7.9	0.58 (0.18)	0.70 (0.19)	0.18*	14	1
	*Group 3*	*Fsi*‐3	40	6.3 (2.4)	6.1	0.54 (0.18)	0.64 (0.18)	0.15*	3	4
	*Group 4*	*Fsi*‐4	214	10.2 (3.0)	7.7	0.60 (0.19)	0.70 (0.20)	0.16*	21	23
	*Group 5*	*Fsi*‐5	82	9.7 (2.8)	8.7	0.59 (0.18)	0.75 (0.19)	0.19*	16	1

The European wildcats were clustered into: group 1 (north‐eastern Alps, Dinaric Alps, Bulgary, and Poland; *Fsi*‐1); 2 (peninsular Italy, Sicily; *Fsi*‐2); 3 (central Germany; *Fsi*‐3); 4 (south‐western Germany and central Europe including Belgium, Switzerland, and Luxembourg; *Fsi*‐4); 5 (Portugal, Spain; *Fsi*‐5). All putative hybrids and two introgressed populations (Scotland and Hungary) were excluded. *N* = sample size; *N*_A_ (standard deviations in parenthesis); and *N*_AR_ = mean number of alleles and allelic richness per locus (*N*_AR_ obtained for *n* = 26, the number of African wildcats); *H*_O_ and *H*_E_ = observed and expected heterozygosity (standard errors in parenthesis); *F*_IS_ = inbreeding coefficient (*significant departures from HWE at *P* < 0.001, Bonferroni corrected); HWE and LE = number of loci (HWE) and pairwise correlation tests (LE) out of Hardy–Weinberg and linkage equilibrium.

### Identification of admixed populations and hybrid individuals

**Figure 2:**
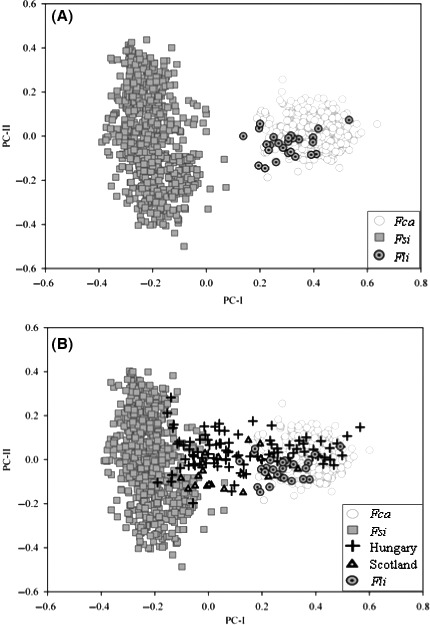
Principal component analysis (PCA) showing the multivariate clustering of the sampled European wildcats (*Fsi*), African wildcats (*Fli*), and domestic cats (*Fca*). The PCA was computed excluding (A) or including (B) the admixed cat populations sampled in Scotland and Hungary. The introgressed cats sampled from the Hungarian and Scottish populations are intermediately dispersed between the wildcats and domestic cats.

**Figure 3:**
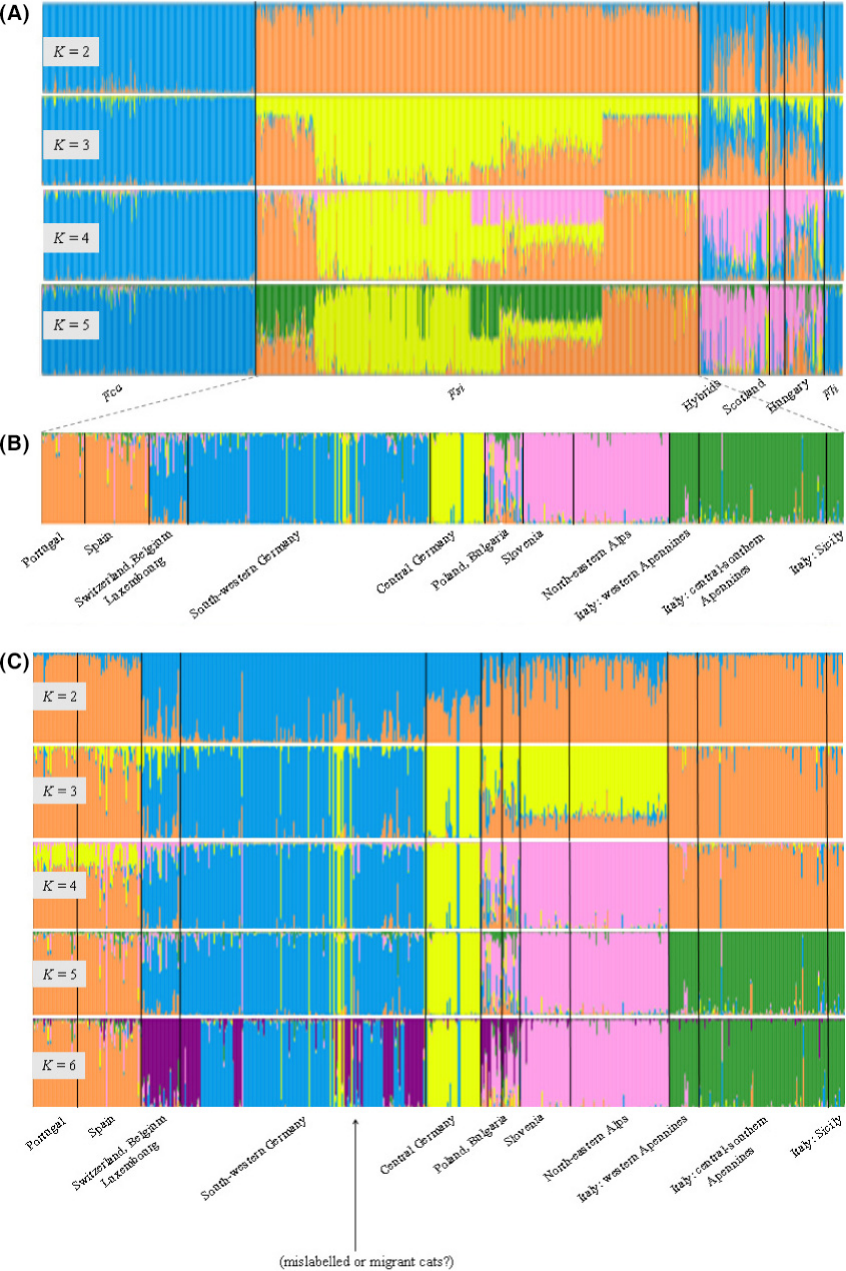
Bayesian clustering analyses of wildcats and domestic cats genotyped with 31 autosomal microsatellite loci. Clustering was performed in structure (run with the “admixture” and the “*F”* models; Pritchard and Wen 2003). (A) Assuming *K* = 2–5, structure shows a major distinction between domestic and wildcats; hybrids and free‐ranging cats sampled in Hungary and Scotland show deeply admixed genotypes. (B) Population clustering assuming *K* = 5 and showing evidence of five main European wildcat biogeographic groups. (C) Patterns of hierarchical splitting of European wildcat populations assuming *K* = 2–6. Each cat genotype is represented by a vertical bar split in *K* colored sections, according to its relative assignment to the *K* genetic clusters.

The European wildcats and domestic cats (plus the African wildcats) plotted into two distinct clusters in a DAPC computed using the entire sample set (Fig. 2A), with the exception of cats sampled from Scotland and Hungary, which plotted intermediately (Fig. 2B). Structure analyses performed with the “admixture” model and *K* = 1–15 (the largest increase in Δ*K* was obtained with *K* = 2; Table S3; Fig. S1A) confirmed the deep domestic *x* wild admixture in Scottish and Hungarian cats (Fig. 3). Assuming *K* = 2, all the domestic cats and the African wildcats were assigned to the same cluster I (the *Fca* + *Fli* cluster) with average *Q*_Fca_ = 0.968 and *Q*_Fli_ = 0.920, respectively, clearly different from all the European wildcats, which were assigned to cluster II (the *Fsi* cluster) with membership values > 0.920 (Fig. 3A). Wildcats from Portugal showed the lower membership value (*Q*_Fsi_ = 0.925), while wildcats from Germany showed the highest (*Q*_Fsi_ = 0.983; Table S4). In contrast, cat genotypes from Scotland and Hungary were admixed showing intermediate values of *Q*_Fsi_ = 0.515 and 0.405, respectively (Fig. 3A; Table S4). Individual assignments were frequently intermediate, with as much as 66.66% (14 out of 21 samples in Scotland) and 83.67% (82 out of 98 in Hungary) of the samples showing *q*_*i*_ values between 0.20 and 0.80. At threshold *q*_i_ = 0.80, we identified 77 admixed samples in the European wildcat populations, including one misclassified domestic cat, seven captive‐bred hybrids and ten previously identified hybrids (Pierpaoli et al. 2003; Lecis et al. 2006). At *K* varying from 3 to 5, the European wildcat populations were gradually assigned to distinct clusters (Fig. 3A). In contrast, the domestic cats and African wildcats remained assigned to the same cluster, suggesting that genetic divergence among European wildcats populations was larger than between domestic cats and African wildcats. The cats from Scotland and Hungary continued to show evidences of deep admixture also at *K* > 2. All samples with hybrid ancestry were excluded for the further phylogeographic analyses and will be analyzed in another study. In this study, we did not further evaluate the admixture in the African wildcats.

### Population structuring in the European wildcats

**Figure 4:**
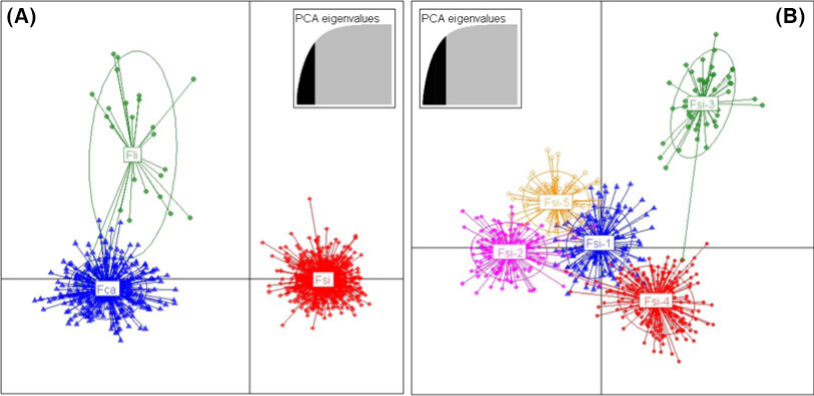
Discriminant analysis of principal components (DAPC in Adegenet; Jombart et al. 2008). The plots show the clustering patterns of: (A) three *Felis silvestris* subspecies: Fsi, European wildcat (*F. s. silvestris*); Fca, domestic cats (*F. s. catus*); Fli, African wildcats (*F. s. libyca*); and (B) five European wildcats biogeographic groups identified by Bayesian analyses: *Fsi*‐1, north‐eastern Alps, Dinaric Alps, Bulgaria, and Poland; *Fsi*‐2 peninsular Italy, Sicily; *Fsi*‐3, central Germany; *Fsi*‐4, south‐western Germany; central Europe including Belgium, Switzerland and Luxembourg; *Fsi*‐5, Portugal, Spain. Individuals (dots) and populations (colored ellipses) are plotted within the orthogonal space defined by the first two PCA eigenvalues (inserts).

Hierarchical Structure analyses of European wildcat populations (computed with the “admixture” model assuming *K* = 1 to 15, *popinfo* = 0, “*F*” or “*I*” models, admixed genotypes excluded) revealed the presence of 5–6 main clusters (Fig. S1B) showing that: (1) at *K* = 2, European wildcats sampled in central Europe (south‐western Germany, Belgium, Luxembourg, and Switzerland) clustered separately from all the other samples; (2) at *K* = 3, the samples from central Germany and from south‐western Germany (plus Belgium, Luxembourg, and Switzerland) were assigned to distinct clusters; (3) at *K* = 4, the samples from the Italian north‐eastern Alps and Slovenia were assigned to the same distinct cluster; (4) at *K* = 5, the samples from the Iberian and the Italian peninsulas were split into two distinct clusters; (5) at *K* = 6, the samples from Belgium, Luxembourg, and Switzerland joined their own cluster (Fig. 3B and C). The different runs from Structure provided the same results, and thus, they were combined with Distruct. An exception was for Sicily, which appears as a distinct group only in some runs (see: Fig. S2). However, across the *K* values, we observed some inconsistent individual assignments, for example, some cats sampled in south‐western Germany that were genetically assigned to the central German population. Moreover, the cats sampled in eastern Europe (Poland and Bulgaria) showed persistent signals of admixture with different population clusters. Thus, the most stable pattern of population structuring supported a partition of the European wildcats into five main biogeographic clusters: *Fsi*‐1 (eastern and Dinaric Alps) *Fsi*‐2 (Italian peninsula and Sicily), *Fsi*‐3 (central Germany), *Fsi*‐4 (Belgium, Luxembourg, Switzerland, and south‐western Germany), and *Fsi*‐5 (Iberian Peninsula). Because of the low sample size in some regions, we did not explore evidences of further substructure, although Structure results suggest that local populations could be genetically subdivided at smaller geographical scale. For instance, the European wildcats from Sicily were assigned to a distinct cluster in 1 of 4 replicates at *K* = 6 (Fig. S2), in 2 of 4 replicates at *K* = 8, and at 3 of 4 replicates at *K* ≥ 9. We observed the same subdivision in five population clusters in nonmodel DAPC, computed excluding the admixed cats, which showed that (1) the three cat subspecies are genetically differentiated (Fig. 4A); (2) the African wildcats and the domestic cats plot closely, as expected from their known phylogenetic history (Fig. 4A); (3) the geographical populations of European wildcat clustered into five groups (Fig. 4B), corresponding to the five clusters identified by Structure (these results are detailed in Tables S3B and S5).

### Genetic diversity in the five European wildcat biogeographic groups

The total genetic variability was significantly partitioned among the three cat subspecies (*ф*_ST_ = 0.159; *F*_ST_ = 0.068; *R*_ST_ = 0.392) and among the five European wildcats biogeographic groups (*ф*_ST_ = 0.103; *F*_ST_ = 0.108; *R*_ST_ = 0.143; AMOVA; all *ф*_ST_ values highly significant with *P* < 0.001). A substantial proportion of genetic variation was attributed to mutations (as measured by *R*_ST_) especially when comparing the three cat subspecies: the *r*_st_/*f*_st_ ratio was = 5.8 among subspecies, and 1.3 among the European wildcat biogeographic groups. Divergence between African wildcats and domestic cats (*ф*_ST_ = 0.077; *R*_ST_ = 0.058) was smaller than between African and European wildcats (*ф*_ST_ = 0.163–0.258; *R*_ST_ = 0.051–0.178; Table 2). Pairwise *ф*_ST_ and *R*_ST_ estimates revealed significant partitions of the genetic variability among the five European wildcat groups with *φ*_ST_ values varying from 0.08 to 0.16 (Table 2). The wildcat population in central Germany showed the lowest genetic diversity (*Fsi*‐3), in comparison with the other European wildcat groups. There were no significant differences in genetic diversity among the remaining European wildcat populations (Table 1). All the five European wildcat population clusters showed significant positive *F*_IS_ values (*P *< 0.001), suggesting population substructuring. However, the number of loci out of HWE within the clusters was smaller than in the pooled European wildcat sample, supporting the population substructure (Table 1). The number of significant pairwise correlations among loci was 81 in the total *Fsi* sample, a likely consequence of nonrandom matings in domestic cats, but it was lower in the wildcat groups (Table 1).

**Table 2:** Genetic divergence (*φ*_ST_ below the diagonal; *R*_ST_ above the diagonal) computed at 31 autosomal microsatellites for pairwise comparison between domestic cats (*Fca*), African wildcats (*Fli*), and five European wildcat biogeographic groups (*Fsi*)

	*Fca*	*Fsi*‐1	*Fsi*‐2	*Fsi*‐3	*Fsi*‐4	*Fsi*‐5	*Fli*
*Fca*		0.045	0.023	0.110	0.026	0.034	0.058
*Fsi*‐1	0.196		0.029	0.050	0.021	0.052	0.100
*Fsi*‐2	0.183	0.103		0.137	0.014	0.048	0.106
*Fsi*‐3	0.217	0.142	0.163		0.100	0.106	0.178
*Fsi*‐4	0.184	0.076	0.112	0.123		0.047	0.109
*Fsi*‐5	0.169	0.089	0.080	0.133	0.109		0.051
*Fli*	0.077	0.220	0.202	0.258	0.206	0.163	

### Inference of past demographic changes in European wildcat populations

**Figure 5:**
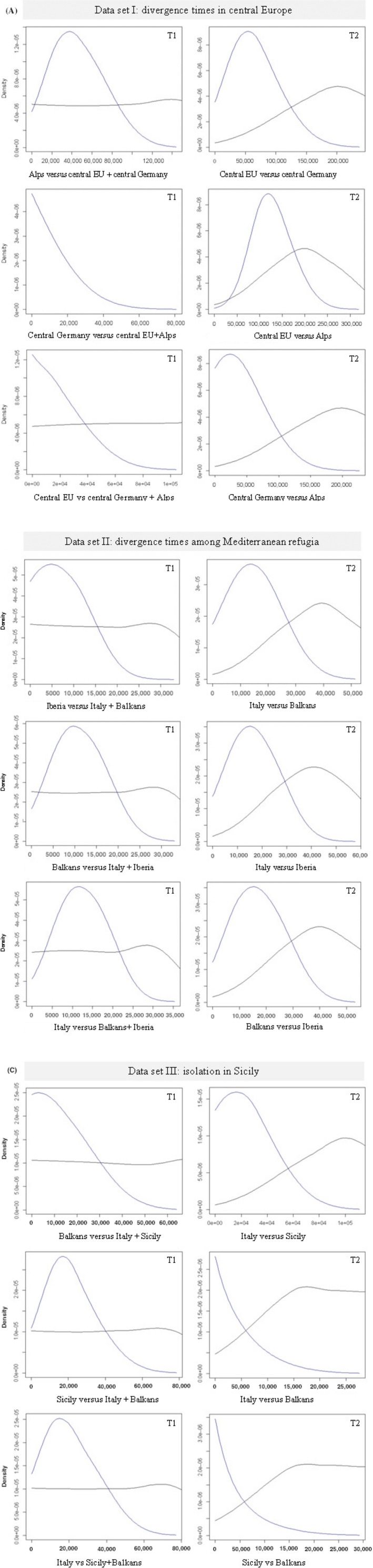
Prior (straight, darker) and posterior (bell‐shaped, lighter) distributions of divergence times (T1, T2) estimated by popABC (Lopes et al. 2009) in three set of European wildcat samples assuming three demographic scenarios (i.e., scenarios 1, 2, 3; see Fig. S3). *X*‐axis = years; *Y*‐axis = density values of T estimates. Estimates of divergence times are determined in: (A) central European wildcats, among samples collected in central Germany, central Europe (Belgium, Luxembourg, Switzerland, south‐western Germany), and Alps (Italian north‐eastern Alps and Dinaric Alps); (B) wildcats likely originating in the Mediterranean refugia of Iberian Peninsula (Portugal and Spain), Italian Peninsula (western and central‐southern Apennines, Sicily), and in the Balkans; (C) Sicily, among wildcat samples collected in Italian peninsula, Sicily, and Alps.

The model values and the 0.25–0.75 quantiles of the posterior distributions for divergence times (T1 and T2) among the five population clusters, estimated using the popABC procedure, are shown in Table 3. Four phylogeographic models yield negative values of posterior distribution parameters (Fig. 5A, scenario 2 and 3; C scenario 2 and 3; Fig. S3) and negative modal values of divergence times in datasets I and III (Table 3), indicating poor fitting of the data to these models. In all the other dataset/model combinations, the posterior distribution of T1 and T2 was bell‐shaped (Fig. 5). The posterior modal values ranged from T2 = 13,000 to 125,000 years, and from T1 = 5000 to 41,000 years. The Alps–central Germany–central Europe populations showed the highest divergence times (T2 = 21,000–125,000 years). The Iberian–Italian–Alps populations showed the lowest divergence times (T2 = 14,000–16,000 years). The isolation of European wildcats in Sicily has been dated approximately at T = 13,000 years. In every case, the uncertainty of the modal values was high, as shown by the 0.25–0.75 quantiles (Table 3). The divergence times computed from the microsatellite genetic distance (*δμ*)^2^ calibrated by mutation rates *μ* = 5.6 × 10^−4^ or *μ* = 2.05 × 10^−4^ were roughly in agreement with the ABC estimates (Table 4). We did not find evidences of recent bottlenecks in the European wildcat groups, with loci in mutation‐drift equilibrium under the TPM model. The *m*‐ratio test showed instead signatures of less recent bottlenecks in wildcats assigned to all biogeographic clusters, with the exception of the European wildcats sampled in Iberia (Table 5).

**Table 3:** Summary of prior distribution parameters, mode, 0.25 and 0.75 quantiles of posterior distributions, and divergence time values estimated using popABC (Lopes et al. 2009) for the European wildcat dataset I, II, and III under three different evolutionary scenarios. The three datasets include (I) samples from central Germany, central Europe (Belgium, Luxembourg, Switzerland, south‐western Germany), and Alps (north‐eastern and Dinaric Alps; (II) samples from Iberian (Portugal and Spain) and Italian (western and central‐southern Apennines, Sicily) peninsula and Alps; (III) cats collected in Italian peninsula, Sicily, and Alps

Dataset	Scenario	Time	Description	Prior distributions	Posterior distributions		
					Mode	0.25	0.75
I	1: ((1,2),3)	T1	Alps versus central EU + central Germany	Uniform (100–200,000)	41,613	21,796	61,662
		T2	Central EU versus central Germany	Uniform (100–200,000)	56,301	26,336	86,775
	2: ((1,3),2)	T1	Central Germany versus central EU + Alps	Uniform (100–200,000)	na	na	na
		T2	Central EU versus Alps	Uniform (100–200,000)	124,996	94,646	156,475
	3: ((2,3),1)	T1	Central EU versus central Germany + Alps	Uniform (100–200,000)	na	na	na
		T2	Alps versus central Germany	Uniform (100–200,000)	21,279	na	51,441
II	1: ((1,2),3)	T1	Iberia versus Italy + Alps	Uniform (0–40,000)	5534	722	10,229
		T2	Italy versus Alps	Uniform (0–40,000)	13,727	6421	21,004
	2: ((1,3),2)	T1	Alps versus Italy + Iberia	Uniform (0–40,000)	10,402	5855	14,925
		T2	Italy versus Iberia	Uniform (0–40,000)	15,447	7722	23,101
	3: ((2,3),1)	T1	Italy versus Alps + Iberia	Uniform (0–40,000)	12,116	7377	16,823
		T2	Alps versus Iberia	Uniform (0–40,000)	15,889	8271	23,482
III	1: ((1,2),3)	T1	Alps versus Italy + Sicily	Uniform (100–200,000)	2665	na	1113
		T2	Italy versus Sicily	Uniform (100–200,000)	13,252	na	29,679
	2: ((1,3),2)	T1	Sicily versus Italy + Alps	Uniform (100–200,000)	18,012	8481	27,767
		T2	Italy versus Alps	Uniform (100–200,000)	na	na	na
	3: ((2,3),1)	T1	Italy versus Sicily + Alps	Uniform (100–200,000)	16,782	6016	27,560
		T2	Sicily versus Alps	Uniform (100‐200,000)	na	na	na

na, negative values of posterior distribution parameters.

**Table 4:** Estimated divergence times (years) among the five European wildcat biogeographic groups computed using the microsatellite genetic distance *δμ* ^2^ (Goldstein et al. 1995b), and two microsatellite mutation rates: *μ* = 5.60 × 10^−4^ (below the diagonal) and *μ* = 2.05 × 10^−4^ (above the diagonal)

	Central Europe	Central Germany	Alps
Central Europe	–	31,221	277,921
Central Germany	114,290	–	279,762
Alps	101,739	102,413	–

	Alps	Apennines	Sicily
Alps	–	61,179	63,756
Apennines	22,396	–	54,701
Sicily	23,339	20,025	–

Samples from central Europe include wildcats collected in Belgium, Luxembourg, Switzerland and south‐western Germany; while Alps regroups cats sampled in Italian north‐eastern Alps, Slovenia, Austria and Bosnia‐Herzegovina. Moreover, all cats collected in the Italian western and central‐southern Apennines were indicated as Apennines.

**Table 5:** Bottleneck signatures in five European wildcat biogeographical groups estimated using the M‐Ratio (Garza and Williamson 2001) and Bottleneck (Cornuet and Luikart 1997) procedures computed assuming 90% stepwise mutations

Populations	Acronym	*N*	M‐Ratio					Bottleneck
			M	Critical m (*θ* = 5)	Average M (*θ* = 5)	Critical m (*θ* = 10)	Average M (*θ* = 10)	*P *< 0.05
*Group 1*	*Fsi*‐1	112	0.737	0.775	0.827	0.768	0.815	1.000
*Group 2*	*Fsi*‐2	132	0.746	0.779	0.829	0.772	0.819	1.000
*Group 3*	*Fsi*‐3	40	0.696	0.753	0.809	0.732	0.784	0.992
*Group 4*	*Fsi*‐4	214	0.755	0.782	0.833	0.782	0.827	1.000
*Group 5*	*Fsi*‐5	82	0.796	0.771	0.823	0.760	0.808	1.000

## Discussion

Sound conservation plans should be based on robust knowledge of species’ biology, distributions, population genetic structure, and dynamics, which are still missing for the European wildcat. We planned this study to reconstruct a first framework of European wildcat phylogeographic structure, aiming at delimiting evolutionary and management units for conservation planning. We hypothesized that the extant patterns of genetic structuring of European wildcat populations distributed in the central and south‐western regions of the continents should have been mainly determined by late Pleistocene climatic changes rather than by recent anthropogenic habitat fragmentation. Our results support this hypothesis.

The studied populations of European wildcat are geographically structured and present relative high levels of genetic diversity. Model‐based structure analyses and nonmodel multivariate clustering concordantly indicate that the sampled European wildcat populations are subdivided into five main genetic clusters showing congruent geographical distributions. Results of ABC simulations and calibrated genetic distances suggest that the main phylogeographic splittings among European wildcat populations were the consequences of late Pleistocene events, and not of very recent anthropogenic fragmentation. However, recent fragmentations could have eroded the within‐cluster genetic diversity, leaving signatures of bottlenecks in all clusters except the European wildcats samples in Iberia. We identified wild × domestic cat hybrids across the entire distribution in Europe. However, hybrid prevalence and introgression depth vary severely among the different countries (indicate range). Wild‐living cats in Scotland and Hungary are deeply introgressed, making difficult the identification of pure parental cats, as previously described using smaller STR panels and cat sample sizes (Pierpaoli et al. 2003; Lecis et al. 2006), or using SNPs (Oliveira et al. 2015). In contrast, European wildcats and domestic cats sampled from the other European countries are genetically distinct, although we identified from *c*. 5% to 10% putative hybrid individuals in the Iberian and Italian peninsulas and Germany.

### Phylogeographic structure of European wildcat populations

Our results allow, for the first time, to assess the European wildcat phylogeographic structuring across the entire species’ range in the continent. The European wildcats in Continental Europe belong to at least five major phylogeographic groups. This partition confirms and strengthens findings previously reported by Pierpaoli et al. (2003). These authors described a main genetic subdivision among the European wildcat populations distributed in southern and central Europe and separated the wildcats in central Germany from all the other European populations. In our study, we identified additional subdivisions. In particular, we showed that wildcats in southern Europe are differentiated in two deeply divergent groups: Iberia (Portugal and Spain) and Italy. At a smaller geographic scale, wildcats in peninsular Italy are differentiated into three genetic groups coherently distributed in Sicily, peninsular Italy, and the Alps (Mattucci et al. 2013), suggesting distinct phylogeographic histories. Moreover, we showed that wildcats in the Italian and Dinaric Alps (Slovenia and Croatia) joined into a unique genetic cluster, indicating recent shared ancestry.

This phylogeographic pattern fits well to a model of late Pleistocene isolation and genetic diversification of European wildcat populations into three main Mediterranean glacial refuges in the southern Iberian, Italian, and Balkan peninsulas (Hewitt 1999). Estimated divergence times indicate that genetic diversity among the five phylogeographic groups has been likely generated during the Late Pleistocene. Based on divergence dates, we can exclude that the observed pattern of population fragmentation arose in consequences of recent anthropogenic pressures. Instead, our results suggest that protracted isolation before the end of the Last Glacial Maximum, originated three well‐differentiated European wildcat populations in the Iberian peninsula, Italian Apennines (and Sicily), and the northern Balkans, around 21,000–125,000 years ago. The postglacial wildcat expansion from a not yet identified Balkan refuge led to the recolonization of the Dinaric and Italian Alps, and originated populations that share their most recent genetic ancestry. These populations are still demographically connected in the northern part of their current distribution (eastern Italian Alps, Slovenia, Croatia). The estimated time of the European wildcat isolation in Sicily (13,000 years ago) is in agreement with known late Pleistocene–early Holocene climate changes and consequent Mediterranean Sea level fluctuations (Magny et al. 2007). We cannot exclude more recent small‐scale subdivisions and ongoing processes of local adaptation as the ones described in wildcat populations distributed in the central Italian Apennines (Mattucci et al. 2013). European wildcat populations living in broadleaved forests in the core areas of their distributions, and those populations living in peripheral Mediterranean habitats in south‐western Iberia and Italy, certainly experience different climate, habitat, and prey community conditions, perhaps promoting divergent local adaptations.

The consequences of climate changes were partially species‐specific, depending on preglacial species distributions, local topographic features, adaptations, and ecological flexibility (Stewart et al. 2010). However, the description of some generalized patterns, including the identification of three main Mediterranean refuges, prevalent postglacial recolonization routes and predicted patterns of geographical variation of population genetic diversity, are being used to describe cryptic *taxa* and identify evolutionary and conservation units (Funk et al. 2012). The inferred European wildcat phylogeographic framework is congruent with many other reconstructions in mammalian species in Europe. The location of glacial refuge areas and the directions of postglacial dispersal routes, although in part species‐specific, are roughly congruent in brown bear (*Ursus arctos*), wolf (*Canis lupus*), red deer (*Cervus elaphus*), roe deer (*Capreolus capreolus*), wild boar (*Sus scrofa*), chamois (*Rupicapra rupicapra*), and in wildcats (*Felis silvestris*) (Schaschl et al. 2003; Pilot et al. 2006; Scandura et al. 2008; Sommer et al. 2009; Davison et al. 2011; Mattucci et al. 2013). Phylogenetic and paleontological findings pointed out to an eastern origin of the ancestral European wildcat populations, which dispersed northward in Europe at least since 130,000 years ago (Sommer and Benecke 2006), following divergence from the African wildcat sister species, *c*. 200,000 years ago years ago (Driscoll et al. 2007). Initial and perhaps replicated east‐to‐west mid‐Pleistocene dispersal waves of ancestral wildcat populations (Randi 2007), could have originated the refugial populations in the three Mediterranean peninsulas. More exhaustive analyses within each of the phylogeographic groups could reveal local subpopulation structuring (such as refuges‐within‐refuge) or undetected areas of wild × domestic cat admixture. Further details at smaller geographical scale will predictably refine this phylogeographic framework, which could be integrated with climate and habitat data. Landscape genetic analyses may lead to describe patterns of gene flow across ecological corridors and eventually identify local adaptations (Joost et al. 2013).

### Genetic admixture, hybridization, and introgression in cats

All the microsatellite loci used to assay the genetic variability are polymorphic in the three sampled cat subspecies: the European wildcat, the African wildcat, and the domestic cat, as demonstrated by allelic richness and observed heterozygosity. The multilocus genotypes show lower than expected heterozygosity and significantly positive *F*_IS_ values when pooled within each subspecies. Deficiency of heterozygotes, compared to the expected HWE proportions, suggests that the groups (cat subspecies) were mixtures of individuals sampled from genetically distinct natural populations (Wahlund effect; Wahlund 1928), or domestic cat breeds (Lipinski et al. 2008). The individual assignments of the European wildcat genotypes to their geographic clusters were, overall, robust, suggesting that the five groups indeed represent the major genetic subdivisions among wildcats in Europe. However, in each of the five clusters, we observed individual genotypes with signals of genetic admixture. Some of them could have admixed ancestry, could have been originated in other clusters (i.e., they are very recent migrants), or could have been mislabeled during sampling procedures. Additional sampling and population structure analyses at local scales could clarify these issues. Our results also showed evidences of genetic admixture in the easternmost wildcat populations (the cats sampled from Poland and Bulgaria), which could have been generated by recent admixture among unsampled wildcat populations. Additional European wildcat samples from unsampled eastern European regions are needed to improve the phylogeographic framework, to identify eventual eastern refugial populations, postglacial east‐to‐west dispersal routes, and areas of secondary contact in central Europe.

Using a larger microsatellite panel and a more comprehensive number of reference genotypes than in previously published studies (Beaumont et al. 2001; Pierpaoli et al. 2003; Lecis et al. 2006; Oliveira et al. 2008b), we confirmed the domestic × wildcat admixed composition of wild‐living cats in Scotland and Hungary. Molecular and morphological identifications concordantly evidenced the consequences of genetic admixture, which makes it difficult to ascertain whether any pure wildcat is still surviving in these two countries (e.g., Pierpaoli et al. 2003; Kitchener et al. 2005). European wildcat populations in other areas of the continent revealed scanty evidences of hybridization and no deep introgression. The causes of strongly variable introgression rates in different parts of Europe are not known, although historical factors (e.g., eradication of local wildcat populations and rapid expansion of free‐ranging domestic cats in Scotland) or landscape features (e.g., patch of forests intermixed with traditional agricultural fields in Hungary) could have had a role locally. Moreover, backcrossed cats are not easily identified by limited panels of microsatellite markers (Vähä and Primmer 2006), and local rates of introgression could have been underestimated. The use of more informative DNA markers (e.g., ancestral informative SNPs; Nussberger et al. 2013; Oliveira et al. 2015) or variation at domestication genes identified through entire genome analyses (Montague et al. 2014; Tamazian et al. 2014) can potentially improve the detection of admixture ancestry.

Hypervariable microsatellite loci are still the markers of choice used to detect fine‐scale population structuring and estimate population genetic variability (Queiros et al. 2014). However, microsatellites have their drawbacks when used to describe not‐so‐recent evolutionary events. Reliable modeling of microsatellite evolutionary dynamics is crucial particularly to evaluate the impact of homoplastic mutations in analyses of population divergence and phylogeography. Microsatellite mutation mechanisms are complex and still poorly known (Goldstein et al. 1995b; Slatkin 1995). Variability in DNA replication slippage rates, length constraints, and instability made it uncertain to quantify microsatellites mutation rates and their variations at different divergence times. In this perspective, the divergence times we have estimated by ABC simulations or genetic distance calibrations could have been biased by unknown microsatellites mutation dynamics. Thus, we offer the time frames described in this study as a working hypothesis that could be tested when cat populations will be genotyped with larger panels of informative autosomal markers derived from entire genome sequences (Montague et al. 2014; Tamazian et al. 2014). Sequences from the mtDNA genome have been extensively used to identify maternal phylogenies and phylogeographic patterns (Avise 2009). However, mtDNA phylogenies in cats have been constrained by extensive transfers of mtDNA genes in nuclear chromosomes (numts; Lopez et al. 1996) and by the still unknown occurrence of domestic cat mtDNA introgression into wildcat populations.

### Conservation implications

The wide post‐World War II expansion of broadleaved forests in most of the European countries, the increased number of protected areas, and sustainable use of other forests should contribute to secure the future of European wildcat populations and their micromammal prey communities. However, the ongoing global climate change trends are increasing the rates of desertification in the Mediterranean peripheries of the European wildcat distribution. Mediterranean forests and maquis habitats are exposed to desertification, and their micromammal communities can drastically change in the near future, compromising the long‐term persistence of wildcat populations.

The European wildcat is a protected flagship species of special conservation concern (Driscoll and Nowell 2010). During the last few centuries, anthropogenic habitat fragmentation and direct persecution disrupted the distributions of wildcat populations in most of Europe (Nowell and Jackson 1996). Although recent reports suggest that some populations are locally expanding (Steyer et al. 2013; Velli et al. 2015), the species’ continent‐wide distribution and abundance are still poorly known. Some national protection plans, based on habitat restoration and the reconstruction of ecological corridors, have been activated (Klar et al. 2012). Additional conservation efforts are needed, particularly to mitigate hybridization and risks of introgression, the consequences of the widespread and uncontrolled diffusion of free‐ranging domestic cats (Randi 2008). Sound conservation plans should be based on robust knowledge of species’ biology, distributions, population genetic structure, and dynamics, which are still missing for the European wildcat. The five European wildcat population clusters described in this study show suitable levels of genetic variability. However, we could not exclude that isolated small patches within these groups might have been exposed to the deleterious consequences of genetic drift and inbreeding. Thus, we suggest improving ecological networks and connectivity among population patches within each of the five population clusters. Ecological networks would also facilitate the re‐colonization of areas where the species is now extinct. A widespread network of ecological corridors could help European wildcats survival and sustain their future evolvability by: (1) increasing the rates of gene flow among local isolated small population fragments, thus counteracting the consequences of drift and inbreeding; (2) increasing the genetic effective size of metapopulation networks; and (3) providing migration pathways to escape the ecological consequences of climate changes and desertification. The effective use of corridors and the expansion of wildcat populations should be assessed by continuous monitoring programs based on noninvasive sampling and molecular identifications. Monitoring programs already assessed the presence of previously unknown viable European wildcat populations in Germany (Vogel and Mölich 2013) and in Italy (Velli et al. 2015). However, networks of thin corridors connecting forest patches could generate undesirable edge effects and increase risks of hybridization. Wildcat dispersal through ecological corridors within a matrix of human‐dominated landscapes should be complemented with strict control of free‐ranging domestic cats. A monitoring program, particularly in fragmented landscapes and across corridors, should be used to assess hybridization between wild and domestic cats, which is considered the major threat for the conservation of wildcat genome.

## Data Accessibility

Sample locations and microsatellite data: DRYAD entry doi:10.5061/dryad.kb13m.

## Conflict of Interest

None declared.

## Supporting information


**Table S1**. Size and geographical origin of cat samples belonging to three subspecies of *Felis silvestris* analysed in this study.
**Table S2**. Description of 31 autosomal microsatellite loci used to genotype samples from three subspecies of *Felis silvestris* (*Fca* = domestic cats, *F. s. catus*;* Fsi* = European wildcats, *F. s. silvestris*;* Fli* = African wildcat, *F. s. libyca*).
**Table S3**. Values of the mean *Ln* posterior probability (Mean lnPD) and maximum lnPD increase (Δ*K*) computed by structure analyses (run with the admixture, independent or correlated allele frequency models and option *popinfo* = 0) assuming a number of *K* clusters variable from 1 to 15, and using: A) three cat subspecies, two introgressed populations sampled in Hungary and Scotland, and putative admixed cats identified in other populations in Europe; and B) only European wild cat samples excluding the two introgressed populations and the 78 putative admixed genotypes identified in A).
**Table S4**. Average proportion membership (*Q*_*i*_) of wildcat populations obtained by structure with *K* = 2, the admixture and the *I* and *F* models, using the three cat subspecies and all the sampled European wildcat populations.
**Table S5**. Average proportion of membership of European wildcat geographical population samples, as determined by structure with *K* values from 2 to 6 (see: Materials and Methods; Fig. 3b).

**Figure S1**. Plot of delta K and mean likelihood L(K) as a function of K averaged over five independent runs of structure run with the ‘admixture and the *F* model’.

**Figure S2**. Evidence of a distinct European wildcat population in Sicily identified by structure with *K* > 6.

**Figure S3**. Demographic histories assumed to estimate divergence times among European wildcat population clusters. Divergence time (not in scale) is reported on the left, ranging from present (T0) to the ancestral population splitting time (T1 and T2).
