# Supplementary material for: European wildcat populations are subdivided into five main biogeographic groups: consequences of Pleistocene climate changes or recent anthropogenic fragmentation?
Source: Ecol Evol. 2015 Dec 7;6(1):3–22. doi: 10.1002/ece3.1815 (PMC4716505; doi:10.1002/ece3.1815)

Mattucci and Oliveira *et al.* Supplementary Figure S1.

Plot of delta K and mean likelihood L(K) as a function of K averaged over five independent runs of STRUCTURE run with the 'admixture and the *F* model'. The Y-error bars are the standard deviation, and K is the assumed number of genetic clusters. Plot A has been obtained using all sampled European wild (*Fsi*) and domestic cats (*Fca*), including the admixed Hungarian and Scottish cats populations. Plot B has been obtained using only the European wildcat populations.

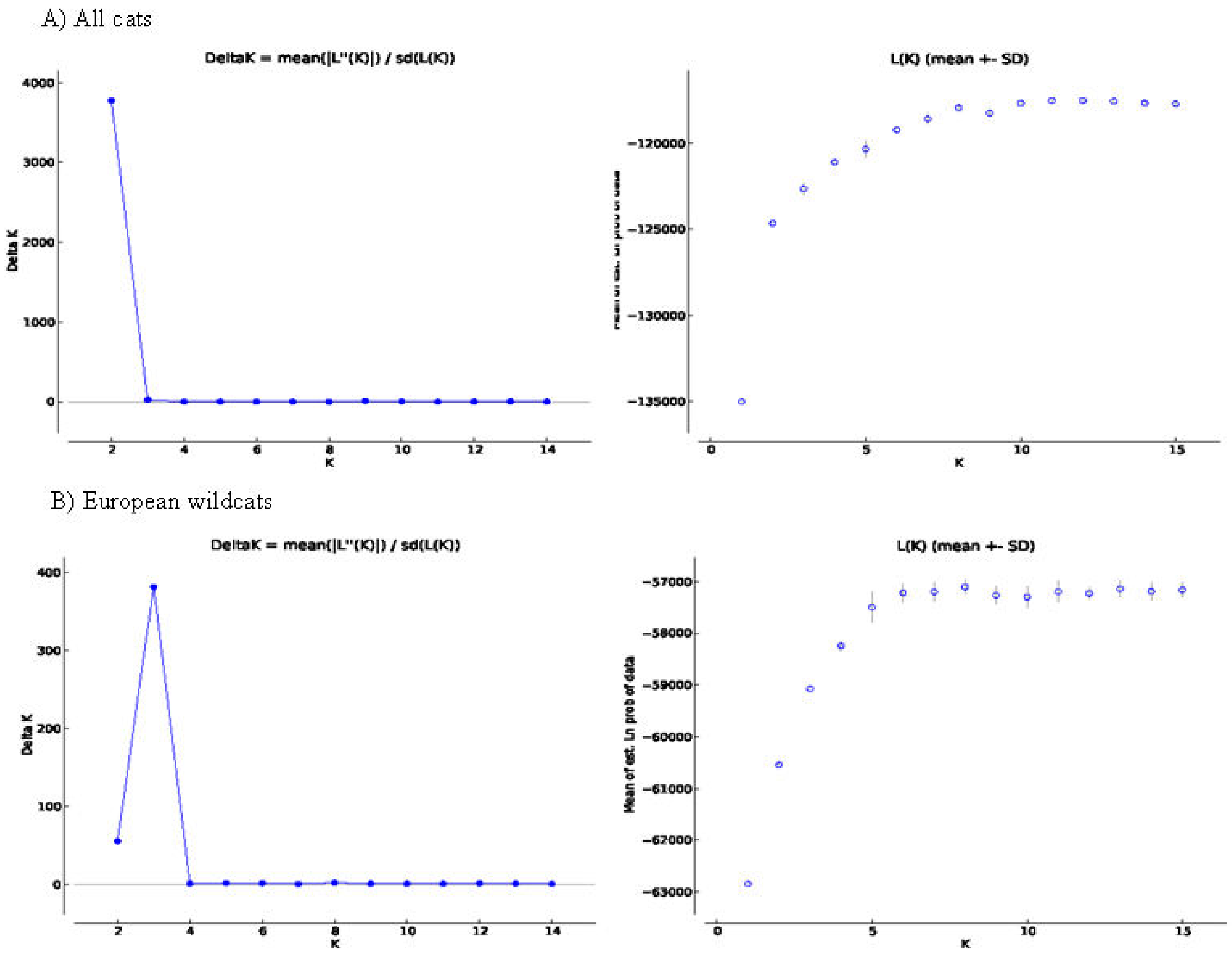

Supplement: Supplementary file 2 — Figure S1. Plot of delta K and mean likelihood L(K) as a function of K averaged over five independent runs of structure run with the ‘admixture and the F model’. [file ECE3-6-003-s002.pdf]
