# Supplementary material for: European wildcat populations are subdivided into five main biogeographic groups: consequences of Pleistocene climate changes or recent anthropogenic fragmentation?
Source: Ecol Evol. 2015 Dec 7;6(1):3–22. doi: 10.1002/ece3.1815 (PMC4716505; doi:10.1002/ece3.1815)

Mattucci and Oliveira *et al.* Supplementary Figure S2.

Evidence of a distinct European wildcat population in Sicily identified by STRUCTURE with $K > 6$.

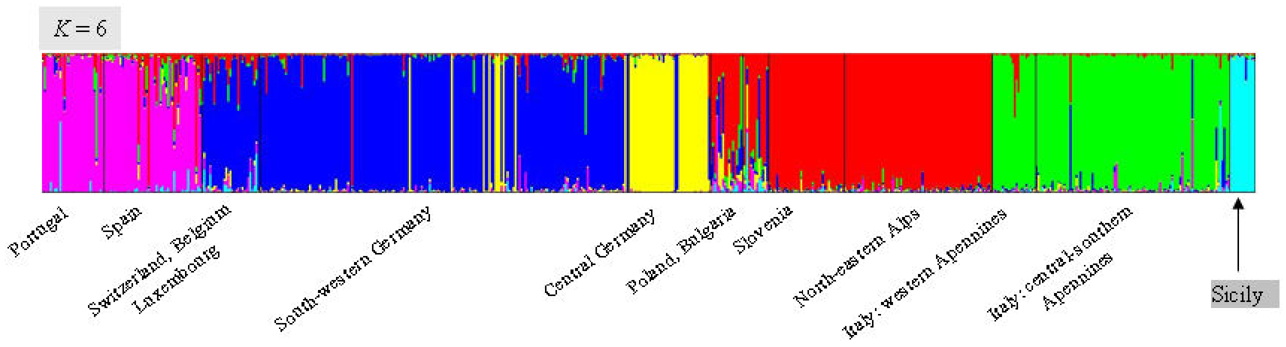

Supplement: Supplementary file 3 — Figure S2. Evidence of a distinct European wildcat population in Sicily identified by structure with K > 6. [file ECE3-6-003-s003.pdf]
