# Supplementary material for: European wildcat populations are subdivided into five main biogeographic groups: consequences of Pleistocene climate changes or recent anthropogenic fragmentation?
Source: Ecol Evol. 2015 Dec 7;6(1):3–22. doi: 10.1002/ece3.1815 (PMC4716505; doi:10.1002/ece3.1815)
Supplement: Supplementary file 4 — Figure S3. Demographic histories assumed to estimate divergence times among European wildcat population clusters. Divergence time (not in scale) is reported on the left, ranging from present (T0) to the ancestral population splitting time (T1 and T2). [file ECE3-6-003-s004.pdf]

Mattucci and Oliveira *et al.* Supplementary Figure S3.

Demographic histories assumed to estimate divergence times among European wildcat population clusters. Divergence time (not in scale) is reported on the left, ranging from present (T0) to the ancestral population splitting time (T1 and T2). Each color represent a theoretical population used to simulate the fourth possible demographic scenarios.

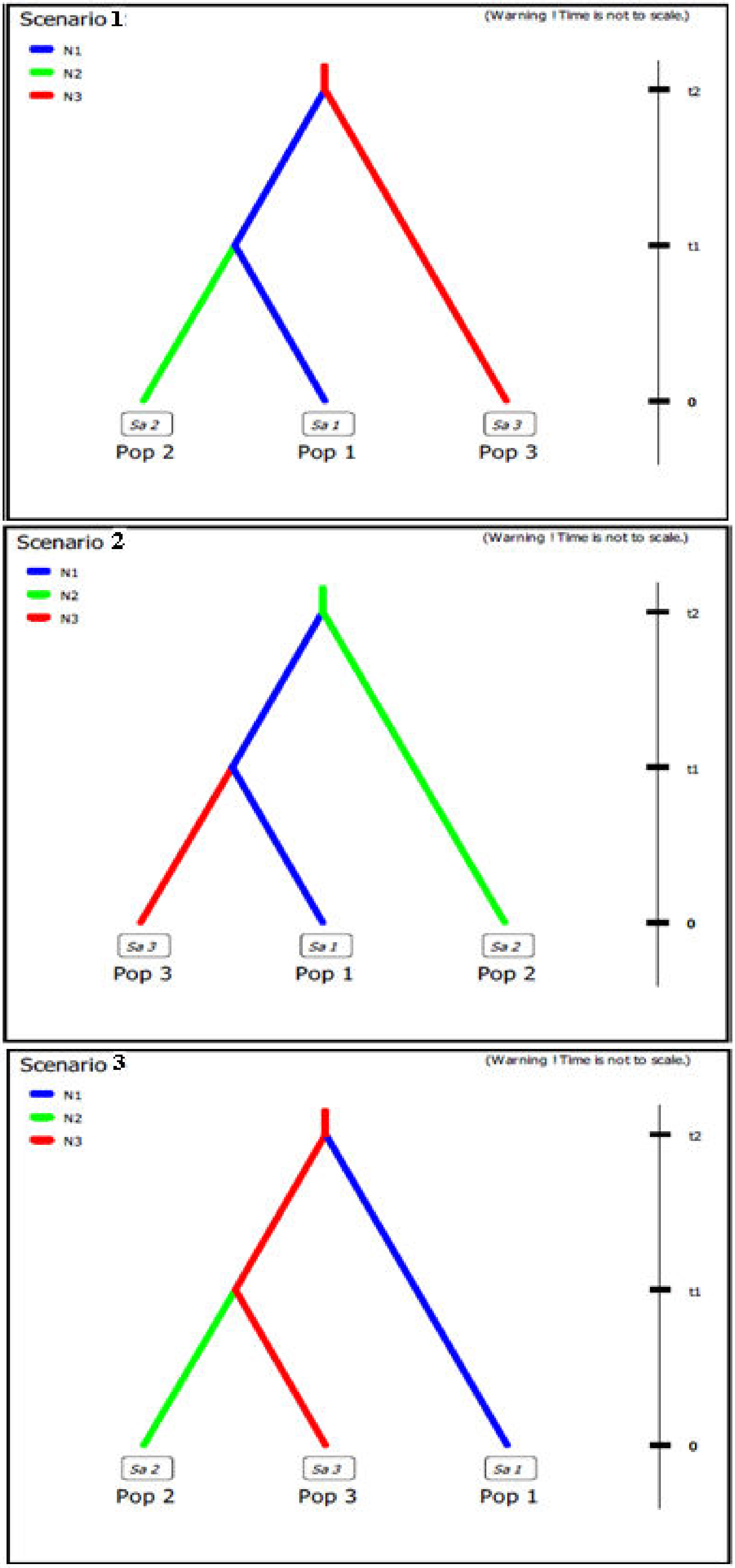
Scenario 1
(Warning ! Time is not to scale.)
N1
N2
N3
t2
t1
0
Sa 2
Sa 1
Sa 3
Pop 2
Pop 1
Pop 3
Scenario 2
(Warning ! Time is not to scale.)
N1
N2
N3
t2
t1
0
Sa 3
Sa 1
Sa 2
Pop 3
Pop 1
Pop 2
Scenario 3
(Warning ! Time is not to scale.)
N1
N2
N3
t2
t1
0
Sa 2
Sa 3
Sa 1
Pop 2
Pop 3
Pop 1
